# Behavior and Bioadhesives: How Bolas Spiders, *Mastophora hutchinsoni*, Catch Moths

**DOI:** 10.3390/insects13121166

**Published:** 2022-12-16

**Authors:** Candido Diaz, John H. Long

**Affiliations:** Biology Department, Vassar College, Poughkeepsie, NY 12604, USA

**Keywords:** biomechanics, spider silk, aggregate glue, Cyrtarachninae, kinematics

## Abstract

**Simple Summary:**

The bolas spider *Mastophora hutchinsoni* creates a small glue droplet attached to a web, called a bolas, which it flicks at a moth flying nearby. When it makes contact with the moth, the glue droplet soaks into the moth’s scales and adheres the moth to the bolas and the spider holding it. Here, we use high-speed video to record the successful capture of moths so that we can understand the physics involved in this system and how the bolas works. In our videos, we found that the moth hovers next to the spider before being caught, minimizing the kinetic energy of the bolas upon impact. This makes the glue droplet’s job much easier, giving it time to spread into the moth’s scales. We noticed during capture that the glue droplet stretched like a spring as it was flicked and when it missed, it sprung back into the shape of a sphere. The glue droplets are incredibly elastic with the ability to stick to dirty surfaces. Studying their diversity, understanding their composition, and measuring their material properties are beneficial for understanding the evolution and creation of bioadhesives.

**Abstract:**

Spiders use various combinations of silks, adhesives, and behaviors to ensnare and trap prey. A common but difficult to catch prey in most spider habitats are moths. They easily escape typical orb-webs because their bodies are covered in sacrificial scales that flake off when in contact with the web’s adhesives. This defense is defeated by spiders of the sub-family of Cyrtarachninae, moth-catching specialists who combine changes in orb-web structure, predatory behavior, and chemistry of the aggregate glue placed in those webs. The most extreme changes in web structure are shown by bolas spiders, who create a solitary capture strand containing only one or two glue droplets at the end of a single thread. They prey on male moths by releasing pheromones to draw them within range of their bolas, which they flick to ensnare the moth. We used a high-speed video camera to capture the behavior of the bolas spider *Mastophora hutchinsoni.* We calculated the kinematics of spiders and moths in the wild to model the physical and mechanical properties of the bolas during prey capture, the behavior of the moth, and how these factors lead to successful prey capture. We created a numerical model to explain the mechanical behavior of the bolas silk during prey capture. Our kinematic analysis shows that the material properties of the aggregate glue bolas of *M. hutchinsoni* are distinct from that of the other previously analyzed moth-specialist, *Cyrtarachne akirai*. The spring-like behavior of the *M. hutchinsoni* bolas suggests it spins a thicker liquid.

## 1. Introduction

Many, but not all, species of spider use silk and varying degrees of adhesion to ensnare and trap prey long enough to envenomate them [1]. Orb-weaving spiders, known for their “wagon-wheel” shaped webs, are generalists in terms of prey, capturing a variety of aerial insects [1]. Many spider families can be distinguished by their prey capture strategies and the silk structures they create [1,2]. The most derived spiders can produce upwards of seven different silks with unique mechanical properties [1,2].

A common prey type that is difficult for most spiders to catch is the moth, since their bodies are covered in sacrificial scales that allow them to easily escape the adhesives of orb-webs [1,3,4]. Their tiny scales are weakly attached to the underlying integument, and they peel off when in contact with the adhesives of most webs [4]. This defensive mechanism works because the adhesives of orb-weaving ecribellate spiders fail to penetrate the superhydrophobic surface of scales presented by the moths [4,5]. However, these defenses have been overcome by one subfamily of spiders, Cyrtarachninae, which have evolved the ability to capture moths [3,4,6,7].

Cyrtarachninae spiders are able to catch moths because of evolutionary changes in the structure of their orb-webs and in the chemistry of their aggregate glue that is placed on those webs [3,4,8,9,10,11]. For example, species of the genus *Cyrtarachne* take the classical orb-web shape and turn it horizontal, replacing short, tight capture threads with long dangling threads [3,4,7,8]. The liquid silk, called aggregate glue, coats the capture threads and has an extremely low viscosity that allows the glue to permeate the surface of scales and spread within the matrix of channels created by the overlapping scales [4,11]. This glue penetrates not only the top layer of scales but also glues them to the cuticle below [4]. As it spreads, this glue hardens and dries, a behavior not seen in the glues of traditional orb-weaving species [4,8,9,11,12]. For this genus and a few others, the ability to catch moths comes with a trade-off in that web spinning is limited to environmental conditions with relative humidity (RH) at or above 80% [8,9,13].

The most extreme changes in the web structure of the Cyrtarachninae moth catchers are shown by the bolas spiders, who create only a single glue droplet at the end of a thread, the bolas [3,14,15,16,17]. This bolas is extremely large, several millimeters in diameter, and contains excess thread coiled within it known as a ‘windlass’ [14,15]. Female bolas spiders prey on male moths by releasing pheromones to draw them close; remarkably, the spiders are able alter the species of moth they are hunting throughout an evening [14,16,17]. When a moth approaches, the spider flicks its bolas, which it dangles from one of its legs, at the prey. While the unique behavior of this prey capture system has been observed in the field, the exact kinematics of the prey capture technique of bolas spiders has not been analyzed biomechanically [15,16,17].

Here, we use a high-speed video camera in the field to observe the kinematics of the capture behavior of *Mastophora hutchinsoni* [18]. By doing this, we hope to understand the physical and mechanical properties of the bolas during prey capture, the behavior of the moth, and how both factors lead to successful prey capture. We also use these videos to create a numerical model to explain the unique viscoelastic behavior of the bolas silk during the capture event. We observed several populations of bolas spiders throughout evenings over a week and attempted to determine if these spiders also have a humidity dependence or limitation for bolas creation.

## 2. Methods

### 2.1. Field Measurements

From 11 to 17 September 2021, the behavior of bolas spiders, *Mastophora hutchinsoni*, was observed and measured at three sites every night on the Maine Farm, University of Kentucky, Lexington, Kentucky. Each location consisted of an isolated tree either within the farmland (38.121163° N, −84.487288° W) or near the fence line directly outside of the farm (38.118291° N, −84.484114° W), (38.123160° N, −84.485876° W). Observations were made between 7 p.m. and 10:30 p.m., when spider activity ended. The order in which sites were visited varied each day. At each tree, which we identified as hackberry, *Celtis* sp., direct visual observations of bolas spiders’ building behavior were recorded for a period from 10–15 min; the number of bolas spiders actively hunting (questing for prey with their front legs or creating a bolas) and the number of bolases created were tallied. During bolas creation, recordings were made of relative humidity and temperature using a hydro-thermometer (Extech model SDL500-NIST SD Logger, Extech, Nashua, NH, USA). In several instances of bolas creation, temperature and humidity were inadvertently not measured and in those cases, hourly local humidity and temperature readings were used (readings from our instrument were found to fall within ±4 %RH of these) [19]. In addition to the time spent censusing behavior, time was spent videotaping active spiders in an attempt to video the prey capture event. We also provide a Supplementary Video (SV1) we recorded from the same location in September 2022 for a different study. It shows *M. hutchinsoni’s* rare trapline prey capture technique.

### 2.2. Kinematics of Prey Capture

Bolas spiders were observed beginning at sundown and as they transitioned from resting, to actively questing (waving their front legs in the air), to creating a bolas. Once the spider had begun to make a bolas, they were videotaped at night with a single Baslar acA1300-60 gmNIR ACE camera (Baslar AG, Ahrensburg, Germany) which was set up perpendicular to the horizontal plane of the spider. Distances between the camera and the spider varied depending on the position of the spider relative to surrounding vegetation. Prey capture events were filmed at 116 fps, the highest speed for the resolution of our camera, using a Fujinon 12.5 mm 2/3″ lens (Fujifilm, Tokyo, Japan). N = 5); this sample size includes four different spiders and five capture attempts. Since most insects and arachnids do not rely on red light for vision, subjects were illuminated using an ABI LED 54 W near-infrared light (880 nm) to provide adequate lighting without impacting the behavior of the spider or moths [1].

**Figure 1:**
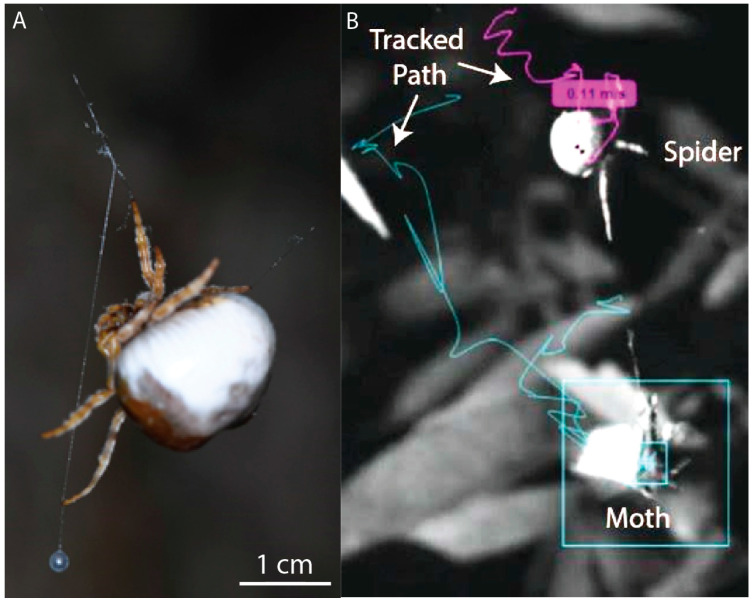
Nighttime, near-infrared image capture of spiders, their bolases, and the moths with which they interact. All images collected in the field. (**A**) *Mastophora hutchinsoni* dangling its bolas, with the large glue droplet, nearly 2 mm in diameter, clearly resolved in this still image. (**B**) The moth approaches the bolas spider slowly in a path (blue) that zigzags, presenting the spider (path in purple) with a target that is close and stable. This example is typical for five of the six of the capture events recorded. It is important to note that the displacements, velocities, and accelerations that we calculated from a single high-speed camera would have underestimated the magnitudes of those properties when the motions moved out of the visual plane that was perpendicular to the camera.

It is important to note that we were capturing three-dimensional movements with a single camera; thus, movements out of the two-dimensional plane perpendicular from the camera resulted in measurements that underestimated the magnitudes of displacements and the velocities and accelerations they were used to derive. At the same time, at night in the field we were able to reliably capture images that resolved the droplet (Figure 1A) and the interactions of the spider and the moth (Figure 1B). From the videos, the movements of the spider, moth, and glue droplet were tracked using a combination of manual and automatic digitizing processes provided by the open-source kinematics program Kinovea (0.8.15) [20] (Figure 1). The position of the spider and moth were tracked beginning just before the moth was caught and until the spider was able to touch the moth with its front legs (N = 5). The software accurately tracked the spider due to its high contrast, but locations were manually verified. The moth was manually tracked, using the head as the focus point, because its fluttering limited the auto-tracking software.

From video images, the diameter of the glue droplet was measured when the droplet was still (Figure 1), and the lengths of the stretched glue droplet and radial capture stretch were measured during flicking (N = 4). A limitation of our videos is that we were only able to see the glue droplet stretch in scattered frames and only from a two-dimensional perspective, as mentioned above. We measured displacements within a two-dimensional plane and used averages over the course of the swing to estimate the forces and velocities. As a first approximation, we assumed that the motion is linear; however, there is an angular momentum to the swing of the bolas, which is likely important to the physics of the overall system but remains unaccounted for here.

The digitized displacements of the spider, bolas, and moth were used to calculate velocities and accelerations of each. Velocities were calculated using finite differences in position and the known time interval between frames. Accelerations were calculated using finite differences in the calculated velocities over the same time intervals. For all values, averages and standard deviations were calculated. Prey falling speed was calculated by measuring the slope of the prey’s position over time, between being hit with the bolas and its freefall being stopped. Using these estimates of velocities and accelerations, impact and kinetic energies of the prey were calculated using the average fresh weight of the moth, 65 mg [16] and our estimate of the mass of the glue droplet from its spherical dimensions and of its density, 1.1 g cm^−3^.

### 2.3. Material Properties of the Droplet

Using the measured stretching of the glue droplet during capture, and the estimated acceleration and mass of the droplet (see previous section), we estimated the glue droplet’s spring constant, *k* (in N m^−1^), and its damping ratio, **ζ**, the ratio of *c,* the damping coefficient, to critical damping, *c_c_*, using a simple first-approximation physics model. We calculated *k* as the ratio of the maximal inertial force and the change in length of the droplet under maximal acceleration. The maximal inertial force of the droplet was estimated from *F = ma_max_,* where *m* is the mass of the droplet (in kg) and *a_max_* is the maximal acceleration of the droplet (ms^−2^) along the path of the swinging thread, as measured from the video. We measured the maximal change in the diameter of the drop, Δ*d*, as it distorted under acceleration; we recognized that as soon as the drop distorted this metric was not strictly a diameter but, rather, the longest linear dimension of the drop.

The Δd, in turn, was used to calculate a spring constant, *k*, for the droplet:

(1) $$k=\frac{ma_{max}}{\Delta d}$$

where *m* is the mass of the droplet. We estimated the mass of the glue droplet as the product of the average resting diameter, *d*, 2 mm (average across all videos) and an estimate of the density, 1.1 g/cm^3^, taken as an intermediate value between the density of water, 1.0, and the density of spider silk threads, 1.3 g/cm^3^ [21].

With an estimate of *k*, we estimated a droplet’s damping ratio, using a dynamic simulation of a linear, one-dimensional mass-spring-damper:

(2) $$F=kd+c\dot{d}+m\ddot{d}$$

where *d* is the sinusoidally-varying diameter of the droplet with the droplet’s velocity, d-dot, acceleration, and d-double-dot, as *d*’s first- and second-order derivatives in a second-order ordinary differential equation. The damping coefficient, *c*, is the unknown parameter (Equation (2)). To estimate the dynamic behavior of the droplet as it is modulated by *c*, we modeled changes in *c* in the dynamic simulation of this mass-spring-damper in the Simulink environment in MATLAB (R2021b) according to established guidelines [22,23]. We varied *c* (μNm^−1^) over three orders of magnitude, 0.1, 1.33, and 10, in order to examine its effect on the deformation of the droplet in three distinct dynamic states: (1) underdamped ζ<<1, (2) critically damped ζ=1, and (3) overdamped ζ>>1 (for customized MATLAB code, see Supplementary Dataset S1). The denominator in ζ, *c_c_,* is the product of 2 and the square root of the ratio of *k* to *m*; *c_c_* was held constant by holding *k* and *m* constant at their average values (Table 1). In each of the three dynamic simulations, we measured the maximum length of the droplet as it stretched; those simulated lengths were compared to measured lengths of the droplet in order to estimate both the dynamic state of actual droplets and the droplets’ value of *c* within an order of magnitude. Strain of the thread and droplet were measured as engineering strain.

## 3. Results

In seven days, from 11 to 17 September 2021, we observed a total of ten spiders at the field site building their bolases. The following behaviors and microhabitat environmental measures were annotated from direct observations. Of those ten, four individuals were recorded capturing moths with high-speed video for a total of five events. Observations, kinematics, and modeling are reported. A single event captured as part of a different study at the same location in September 2022 is reported because it was a different type of bolas-mediated capture.

### 3.1. Observations on Bolas Building Behavior

During sunset, spiders began to move from their hiding spots, which were either on the underside of a leaf or on the top of a leaf with the spider camouflaged with silk splatter. As they emerged from their resting position, spiders would move along branches towards a tip, where they would build their bridge thread between two or more leaf tips or branches. Within the canopy of the tree, spiders chose positions at the crown (outer, near sun leaves) or internally (inner, near shade leaves). Because we had located spiders in their hiding spots during the day, we could determine that most spiders emerged each night; however, some remained in their resting position, sometimes with a single leg held stationary in the air. Spiders were observed from sunset until 10:30 p.m. Some spiders were observed after these times but, with little to no moth activity, those remaining spiders did not create any new bolases.

**Figure 2:**
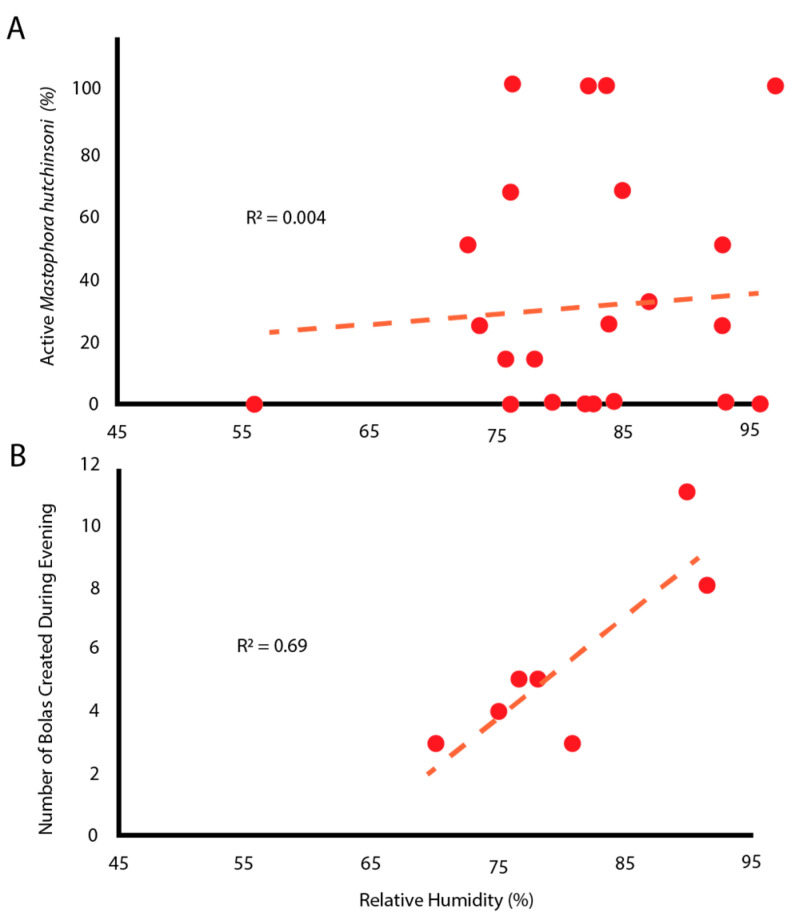
Humidity influenced the making of bolases but not the proportion of spiders who were active. (**A**) Relative humidity did not predict the proportion of spiders that were active. Each point represents observations of multiple spiders on a single tree during a single observation session. (**B**) Relative humidity correlates positively with the number of bolases created. Each point represents all observations on a given day.

Active spiders engaged in four types of behaviors: (1) questing without a bolas, (2) creating a bolas and questing with it, (3) capturing a moth with a bolas, and (4) eating a moth. Spiders were most often found questing without a bolas, actively hanging from a thread with their front legs extended. There was no correlation between the relative humidity level near the tree and the number of active spiders (Figure 2A). However, as relative humidity increased, so too did the number of bolases that were created (Figure 2B). Please note that because individuals were sampled repeatedly, the data points are not independent statistically. Any active individual might not make a bolas, might make only one bolas, or might make multiple bolases in one evening.

**Figure 3:**
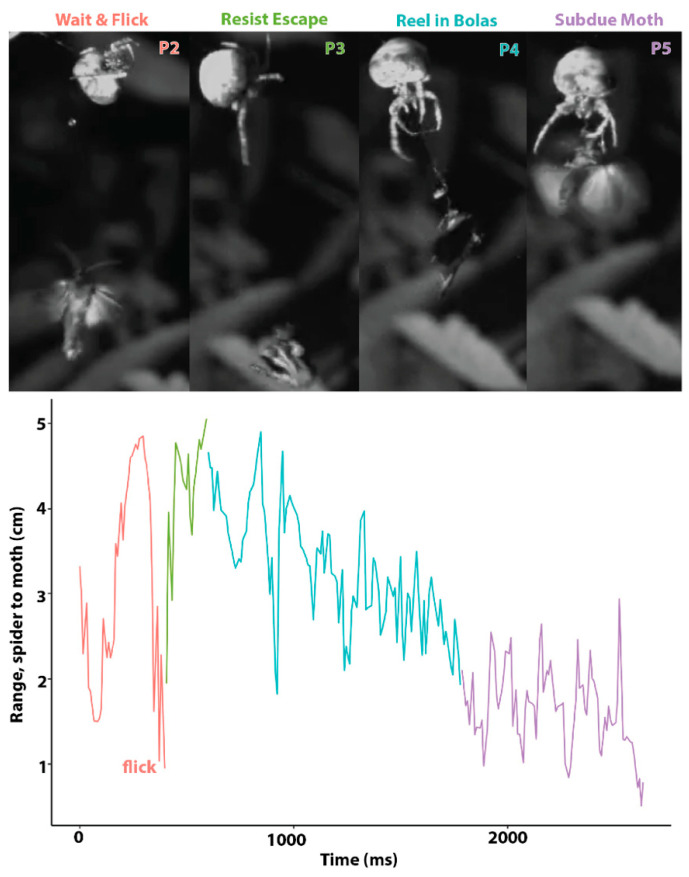
Bolas spider capturing a moth by flicking its bolas. In four of five successful capturing events, the spider and moth interacted in five phases, four of which are shown here (**top**). The distance between the spider and the moth, denoted as range (**bottom**), quantifies the dynamics of the struggle, with the colors of the lines corresponding to the phases above.

In all of the successful moth-capturing events that we observed, the spider used a bolas to capture a bristly cutworm moth, *Lacinipolia renigera*. This does not mean that questing for a moth without a bolas is not a successful strategy, but, rather, that in our limited set of observations we did not see another method. Thus, for five of the six capture events that we observed (the sixth, a different type of bolas-mediated capture, is described in the next paragraph), a successful bolas-flicking moth-capture event can be broken down into five phases (Figure 3), which include (1) creating a bolas, (2) waiting for the moth and flicking the bolas when the moth is close, (3) resisting the escape attempted by the moth, (4) reeling in the bolas with the moth attached, and (5) subduing the moth. Drawn by pheromones produced by the spider, the male moth hovers nearby. The presence of the moth causes the spider to rapidly construct a bolas and then it waits for the next moth to approach before flicking the bolas at it. After being hit by the glue droplet at the end of the bolas, the moth executes an escape maneuver by dropping in free fall. The spider resists the attempted escape by holding onto the radial thread to which the bolas is attached while also holding onto its overhead line; it quickly reels in the attached moth, grasps it, and then subdues it by injecting venom. Reeling in the bolas is the most variable phase, as the moth may attempt to fly while the spider is reeling it in. By measuring the distance between the spider and the moth over time (Figure 3), we were able to calculate a number of kinematic parameters (Table 1).

A dramatically different type of bolas-mediated capture event was observed and recorded once on video (Supplementary Video S1) as part of a different study on spiders at the same location but in 2022; however, it was not further analyzed. After making a single bolas in the usual manner, described above, one spider recycled the unused bolas by eating it and then moved to a new location. There, it created a nearly horizontal trapline approximately 30 cm long, from which it hung three bolases, equally spaced; the spider then moved to the end of the trapline at the higher leaf and waited. This trapline arrangement has been described by [17], but no one, to our knowledge, has observed how the architecture works during moth capture and how the spider behaves when it snares prey. On our video, a moth flew toward the spider and, on a slightly upward trajectory, was ensnared by the middle bolas. The tethered moth began to flap vigorously, spinning around the horizontal thread, causing the horizontal thread to vibrate violently as the spider moved along it toward the origin of the middle bolas. Once it reached the bolas, the spider reeled in the moth. When the moth was in the spider’s grasp, it continued to flap vigorously, spinning the spider around the horizontal thread until it was fully subdued and was still. This trapline behavior differs from that described as the usual method above by substituting the first two stages—(1) creating a bolas and (2) flicking the bolas at the next moth that approaches—with (1) building a trap line, (2) moving to the end of the horizontal thread, and (3) waiting until the moth is ensnared. We also note that the acrobatic walking of the spider along the gyrating horizontal thread is a new behavioral element that is parallel in time with the moth attempting to escape.

**Figure 4:**
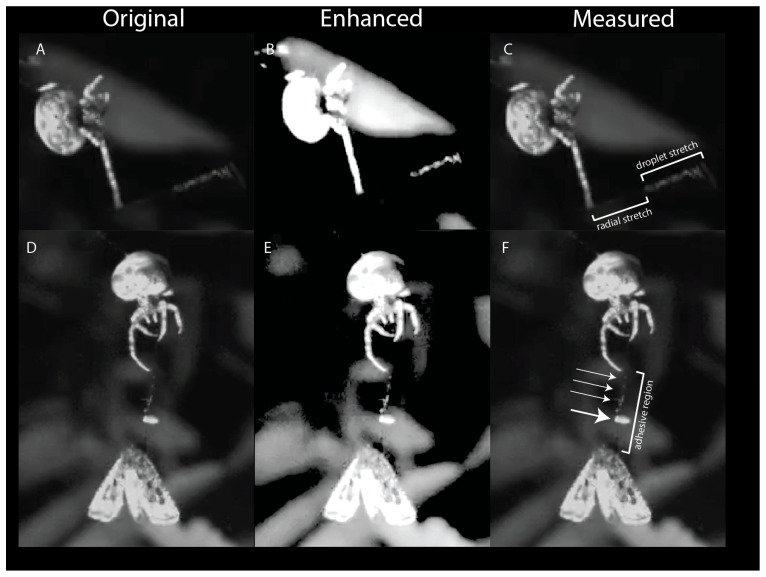
Behavior of the bolas during capture. The spider flicks its bolas towards the moth (off screen to the right). In the original image (**A**), a still from the high-speed video, the bolas can be seen as it stretches. Enhancement of the image (**B**), by increasing the exposure and contrast, more clearly shows the structure of the droplet as it stretches; the liquid phase of the droplet (white) remains associated with the thread of the windlass as it unfurls. The droplet continues to be tethered to the spider by the radial thread, which also stretches (**C**). The spider reels in the moth (**D**,**E**). In the original image (**D**) the spider is hauling in the radial thread as the moth dangles from the stretched droplet. In the enhanced image (**E**), the scales attached to the droplet can be seen as a large clump (elliptical white structure) and smaller clumps (smaller white regions) on the windlass. The stretched droplet forms an elongated adhesive region (**F**), with a large clump (large arrow) and small clumps (small arrows) that were created during the initial deposition of the glue droplet onto the moth.

Each bolas is composed of a radial thread and a glue droplet. The finished bolases, hanging free, have an anchoring thread that remains wrinkled/coiled, not straight, under the weight of the glue droplet. In preparation for flicking the bolas, the spider hangs by one leg from the anchoring thread and places a leg oriented below its body on the bolas near the droplet. The spider will then cock its arm, allowing the bolas to dangle down (Figure 3, ‘Flick’). This is the characteristic posture that we saw in all spiders after they created a bolas and as they awaited the approach of a moth. To flick the bolas towards a nearby moth, the spider rapidly flexed the leg holding the bolas. When the flick was unsuccessful, the droplet would stretch and then recoil. When the flick was successful, attaching the droplet to the moth, the droplet stretched and remained elongated until the moth was subdued (Figure 4A–C). The maximal extent to which glue droplets stretched was, on average, 5.9 times their initial diameter (Figure 4A–C). In addition, the silk thread was also elongated, straining maximally on average 31%, which is within the range of major ampullate thread shown to stretch up to 60% [24].

### 3.2. Kinematics of Prey Capture

Five of six prey capture events, when the spider flicked the bolas (see previous section), were filmed with the bristly cutworm moth, *Lacinipolia renigera*, captured in every case. In discussing the kinematics, it is important to note that we were capturing three-dimensional movements with a single camera; thus, movements out of the two-dimensional plane perpendicular from the camera resulted in measurements that underestimated the magnitudes of displacements and the velocities and accelerations they were used to derive.

The impact velocities and energies of the moths were low because the moths were hovering prior to being struck with a bolas (Table 1). Moths flew extremely close to the spider, within 2 cm, before the spider flicked the bolas. Maximum prey kinetic energy was found during thrashing and not during free fall or impact. The spiders reeled in the moths within three seconds. The maximum impact and falling energies of the moth were low (Table 1) and are observed to be well within the range of energy absorbed by spider capture threads and aggregate glue [2,25,26].

The velocities of the spider and moth varied greatly over time, with the moth having 2.6 times the maximum speed of the spider. The accelerations were highly variable as the moth thrashed and fell, with the highest magnitudes being during the moth’s escape attempt while tethered. The highest velocities were also not during free fall but during escape-related thrashing (Figure 3). The velocity and acceleration of the spider are significantly lower than that of the moth, even though the two are tethered together. This can be attributed to the energy absorption of the bolas thread and the momentum fluctuations dampened by the silk line and leaf the spider is attached to.

### 3.3. Model of Bolas as Viscoelastic Spring during Prey Capture

**Figure 5:**
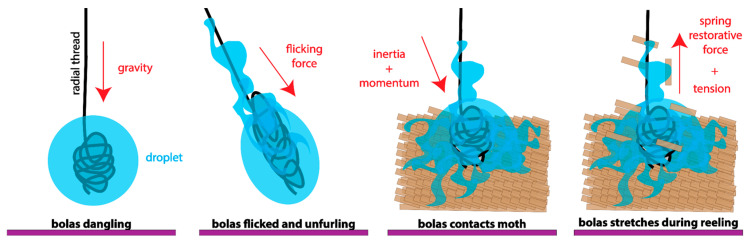
Speculative model of a bolas interacting with the scaled surface of the moth. The bolas has a viscous glue droplet containing a windlass of coiled silk. The flicking force of the spider stretches the glue droplet, with the internal silk of the windlass allowing it to stretch, spring-like, and to retain the liquid phase. If the droplet fails to hit the moth, the glue droplet returns elastically to its original spherical form. If the droplet hits the moth, the collision with the substrate initially dislodges scales. The tension on the thread, caused by the escaping moth, begins to unravel the windlass as it is pulled through the glue, causing the glue to spread within the matrix of the scales. The hydrophobic nature of the scales causes them to be pulled to the surface and pulled upward, cleaning the area and allowing the remaining glue to connect with the underlying cuticle. As the tethered moth struggles to escape, angular momentum is generated, leading to further contact between the glue droplet and moth substrate. The interactions of the bolas, both liquid phase and windlass, with the scales of the moth, are speculative, based on previous work of glue spreading in the previously analyzed moth-specialist, *C. akirai*.

The droplet of the bolas contains within it a coil of silk (the “windlass”) at the center. Videos show that when the spider flicks the bolas at the moth, the mass of the glue droplet creates, by its translational and angular acceleration, a tension force on the thread, which begins to elongate (Figure 4A–C). After the thread has elongated maximally, the droplet begins to deform and reaches its maximum deformation. As the droplet deforms, the windlass inside unravels (Figure 4A–C). In videos where the spider misses its target, the deformation of the droplet is transitionary, and, after peak acceleration, it shortens in a manner that appears to be elastic. This elastic recoil is likely caused by the filamentous windlass. In contrast, the glue droplet does not recoil when the droplet impacts the moth, and the windlass continues to unwind, permitting the droplet to elongate, as the spider reels in the moth (Figure 4D–F). At the interface of the droplet and the moth, the tensile forces dislodge the moth scales and, perhaps because of their superhydrophobicity, they float to the top of the glue droplet and away from the base cuticle (Figure 4D–F and Figure 5). Tethered to the thread, the moth, in thrashing and attempting to fly away, moves itself in a circular path. The circular path of the moth tilts the thread relative to the integument, creating an angular moment at the attachment site that may help to press the glue into the matrix of the scale. This spinning was seen in all capture events recorded on video, and it continued until the spider subdued the moth.

**Figure 6:**
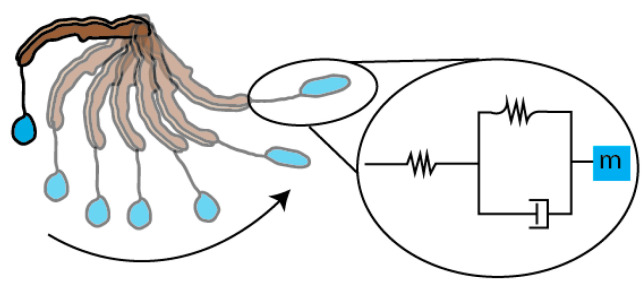
The dynamic behavior of the bolas during flicking, modeled as a simple mass-spring-damper system. The glue droplet is held steady with the leg in a horizontal orientation (top-most position). As the spider swings its leg, the inertia of the flicked glue droplet carries it forward until it reaches the end of its arc. At this moment, the kinetic energy is transduced into elastic energy in the radial thread and the droplet, stretching both (see Figure 4A–C). The system has two springs: (1) the radial thread and (2) the glue droplet, forming the mass-spring-damper system as in the diagram in the inset. The springs are represented by zig-zagged lines, the damper is a piston, and the center of mass is shown by the blue square, *m*. This model is a first-approximation and, thus, is likely to be highly simplified compared to the actual behavior of the system, which has yet to be determined.

Based on the observed dynamics of the glue droplet, we modeled its stretching during the flick as a mass-spring-damper system (Figure 6). Maximum acceleration, $a_{max}$, and spring constant, *k*, estimated from displacements measured by high-speed video and Equation (1), varied in different video observations by a factor of 4 and 3, respectively (Table 2). It is important to note that $a_{max}$ and the *k* that they are used to estimate are likely underestimates, given the limitations of our two-dimensional view of a three-dimensional set of motions (see Methods). Thus, these values of $a_{max}$ and *k* should be treated as rough, preliminary, order-of-magnitude estimates.

**Figure 7:**
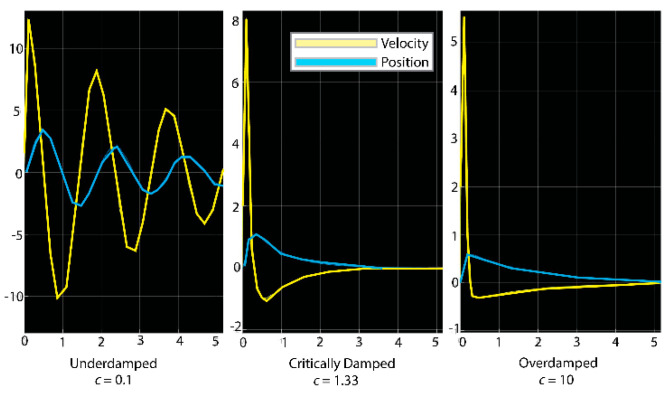
Mass-spring-damper estimates of bolas stretch and velocity. Underdamped ζ<<1; critically damped ζ=1; overdamped ζ>>1. Three models for the damping coefficient resulting in the three types of behavior. Two lines share the common *y*-axis with varying units; yellow lines depict estimated droplet velocity (cm s^−1^) and blue lines the droplet’s stretch from its initial position (cm). The *x*-axis shows time in seconds. The critically damped model yields the behavior that most closely matches actual behavior of the glue droplet. The underdamped one oscillates too much, and the glue droplet stretches too far. The overdamped does not stretch nearly as far as measured.

Using the average *k* of 1.33 μNm^−1^ and average droplet mass of 4.6 mg, we modeled dynamic behavior under three different damping coefficients that were chosen to exhibit vibrations that were underdamped (damping ratio, ζ<<1), critically damped (ζ=1), and overdamped (ζ>>1) (Figure 7). The resulting simulations were used to qualitatively judge the droplet’s actual behavior, determining which model best matched the observed motion. We aimed to determine which vibratory behavior most closely matched the droplet kinematics captured on video. With droplets stretching ~1 cm and rebounding immediately after being thrown, the closest model was the critically damped spring (Figure 7).

## 4. Discussion

To our knowledge, this is the first study to quantify the moth-capturing behavior of the bolas spider, *Mastophora hutchinsoni*. Using a high-speed video camera in the field, we captured the kinematics of the spider’s leg and its bolas—a thread and a glue droplet—as it flicked the bolas and captured a moth. When attached to the struggling moth, the glue droplet undergoes remarkable reconfigurations, stretching to lengths nearly six times that of the droplet’s original diameter. This stretching indicates that the droplet behaves as a viscoelastic spring, as indicated by our first-approximation model of the system as a critically damped mass-spring-damper. This complex mechanical behavior of the droplet is reflected in its complex composition, with a viscous liquid surrounding a coiled thread—which unspools during capture—that is continuous with the dangling thread held by the spider.

Measuring displacements over time to estimate acceleration (Table 2), and knowing the mass of the droplets, we estimated that forces involved in prey capture are relatively low, with kinetic energies of the order of 1.3 μJ. Forces are low because the moths are hovering near the droplet when they are caught; thus, the relative speed of impact of the moth and the droplet are low (Figure 1). This low impact force from hovering moths stands in contrast to the high impact speeds of fast-flying aerial prey caught in orb webs [27]. With the bolas spiders, moths generate the greatest forces after the moth drops to attempt escape and begins to thrash. When their gravity-assisted drop is arrested by the attached bolas and they are tethered, most moths respond by flying, stretching the glue droplet (Figure 4), while the spider works to quickly reel in the moth (Figure 3).

### 4.1. Inquisitive Prey

Male moths approach the bolas because they are attracted to pheromones produced by the spider that mimic those produced by conspecific female moths [3,16,17]. Male moths must first detect the pheromone plume and, then, to find the female, navigate up the plume’s concentration gradient, a behavior that involves a zig-zag flight pattern upwind [28]. This type of chemotaxis, while common in insects, is fickle, requiring wind of low velocity, with little turbulence constant pheromone emission in order for moths to quickly locate the target [29].

However, chemotaxis for the moth is more complicated in most natural conditions, where even low-velocity winds may vary and pheromones may be released in pulses, creating odor gaps in the plume that force the moth to cast, that is, to turn perpendicular to the wind and attempt to recontact the plume [29]. The resulting flight path is slow and tortuous. Thus, as a moth approaches the bolas, it does so slowly, hovering and maneuvering as it searches for the pheromone target (Figure 1). This slow flight presents the spider with a steady target in close proximity. The flick of the bolas, when it comes, meets the moth with a low impact speed dictated more by the length of the bolas and the spider’s leg than the relative speed of the moth and the spider (Figure 3). Thus, the critical first contact between glue and moth is of long duration, allowing the droplet time to permeate the scales and anchor the thread (Figure 5), which are processes that glue the moth’s scales to its underlying integument.

### 4.2. Environmentally Constrained Predators

While specific environmental conditions are required to allow the moth to navigate by chemotaxis, the conditions must also allow the spider to make a glue droplet that stays on the tip of the thread and does not evaporate. Judging from both the activity of the spiders and the number of bolases they create (Figure 2), relative humidities above 75% appear to provide the appropriate hygroscopic balance between evaporation and absorption to allow a large droplet to be created, held in the ready for up to 30 min, and then stay attached to the web as it is flicked towards the moth. In addition, the droplets must also quickly permeate the scales of the moth, glue the scales to the moth’s underlying integument, and then withstand the repeated attempts by the moth to pull free.

The apparent humidity-dependent behavior in *M. hutchinsoni* (Figure 2) may be for different reasons than the environmental constraints that affect droplet formation and mechanical properties in other species of Cyrtarachninae [11,13]. While most orb-weaver spiders make their webs and leave them for the evening, *M. hutchinsoni* glue droplets are relatively short-lived (~30 min) and are recycled by ingestion [15]. In addition, we observed that spiders did not create bolases continuously during a humid evening, nor did they make more bolases after they had successfully captured a moth. More importantly, spiders only made bolases in response to the presence of moths. Thus, moths, which are more active in high humidity [30], may directly trigger the bolas-building behavior of *M. hutchinsoni*. This, then, is an alternative hypothesis to the idea that moth-catching spiders are dependent on high relative humidity for the proper formation and function of their glue droplets [11,13]. These hypotheses may not be mutually exclusive.

In addition to considerations of relative humidity or the presence of moths, the physical structure of the local microhabitat may be important. The activity of spiders varied in ways that may indicate that their location on vegetation has an impact on behavior. Some spiders were inactive or, if active, never made a bolas. The individuals that were found farther away from the branch tips, more towards the trunk of the hackberry trees, responded first to the presence of moths, creating the largest number of bolases. Individuals located on the tips of branches showed less activity. These observations are consistent with the hypothesis that bolas spiders are responsive to variations in the amount of wind in a given tree [16]; while we did not measure wind speed, we conjecture that, in light winds, outer positions may offer the highest probability of attracting and capturing moths, while on evenings of higher winds, the inner positions may shelter or funnel wind in such a way as to allow pheromone plume formation to be coherent enough for chemotaxis by the male moths.

### 4.3. Predator–Prey Interactions via a Viscoelastic Bolas

Once the moth is attached to the bolas, it struggles to escape, putting dynamic loads on the bolas, stretching the droplet to lengths up to five times its original length without breaking (Table 1, Figure 2 and Figure 4). This mechanical behavior of the droplet is remarkable for several reasons. First, the flicked droplet elongates and, if the target is missed, it will recoil; mechanical behavior that is at least partially elastic. Second, during elongation and recoil, the liquid portion of the droplet remains associated with the capture thread, which is a property of material coherence most likely related to the droplet’s internal windlass, a wrapping of thread, continuous with the web from which the droplet dangles, providing attachment surface for the surrounding fluid of the droplet (Figure 5). Third, the dynamics of the droplet’s deformation is consistent with the behavior of a mass-spring-damper system (Figure 6) that is critically damped (Figure 7), suggesting a matching of elastic and dissipative properties in the droplet. Finally, by undergoing extreme elongations as a critically damped elastic system, the droplet attached to the moth has, as a system, dynamic behavior that absorbs and dissipates the energy of the struggling moth. So-called “soft” springs work this way, with their low elastic modulus requiring high strain to generate a high force, slowing the rate of change in the force. What may also be important is that the droplet, by dissipating energy, prevents the moth from generating a high-magnitude jerk, where jerk is the rate of change in acceleration. The viscous dashpot of a shock absorber works this way. Both soft stiffness and high viscosity are accounted for in the mass-spring-damper viscoelastic model (Figure 6).

To help guide future studies, we offer the following prediction that should be tested for its wide phylogenetic claim. We predict that the mechanical behavior of the glue droplet during moth capture—as modeled by a simple mass-spring-damper model—developed here for M. *hutchinsoni* will also apply to the other 50 species of the genus *Mastophora*, the monophyletic taxon of bolas spiders. This prediction is based on the following assumptions: (1) all *Mastophora* species have females that capture moths by flicking a bolas, generating inertial forces in so doing that must be resisted by the droplet in order for it to stay on the web; (2) all *Mastophora* species have a windlass in their glue droplet that provides cohesive forces to retain the glue droplet as it is accelerated; and (3) all *Mastophora* species have a higher viscosity glue droplet than that found in sister taxa within the moth-catching sub-family Cyrtarachninae. These assumptions rest on incomplete data, since only a few species of *Mastophora* have been studied. A more general way to state the prediction is that any spider that flicks a bolas needs that bolas to have elastic and viscous properties that allow the droplet to (1) stay attached to the web, (2) allow the droplet to permeate the hydrophobic scales of the moth, (3) rapidly glue the scales to the underlying integument, and (4) resist the repeated dynamic forces generated by the struggling moth. In whichever form this prediction is stated, it represents the physical requirements for the behavior. Moreover, the first form of the prediction places these physical requirements into the phylogenetic context of the moth-catching specialists in the Cyrtarachninae, a taxon that has evolved a diversity of ways to catch moths, all of which involve correlated changes in web architecture, silk material properties, and behavior [12].

### 4.4. Adaptations for the Capture of Moths

While spiders of the Cyrtarachninae share the evolutionary innovation of catching moths, the different methods for doing so suggest that some key adaptations, shared by the common ancestor of the taxon, has permitted this rapid diversification. Making a lot of glue and having it be able to spread and harden quickly have been proposed as adaptations in the common ancestor [12]. Given the importance of being able to first permeate the super-hydrophobic scales of moths, we suspect that the glue’s viscosity was an important physical property that was altered initially in the taxon’s common ancestor and continued to evolve in concert with the different moth-catching behaviors seen in the extant descendent taxa. Low-viscosity glue is exemplified by that found in *Cyrtarachne akirai*, a species that catches moths with large glue droplets attached to a stationary horizontal web. Upon touching the scales of a moth, the glue droplets quickly permeate the surface of the scales and is spread by capillary forces in the micromesh created by the overlapping scales [4,11]. While in *Mastophora* viscosity has not been measured and spreading studies have not been conducted, the physical requirements mentioned above for flicking a bolas lead us to expect that they may have droplets with higher viscosity than those of *C. akirai*.

*M. hutchinsoni* may use the force of the bolas impacting the moth to spread the glue and force it under the scales, where capillary action can then spread it further—a trait possibly shared by other bolas spiders [31,32]. While each genus of bolas spider varies the structure and behavior of their bolas swing, all rely on the momentum generated by the spider to create impact with their prey. For example, species of the genus *Ordgarius* construct bolas of longer length than *M. hutchinsoni* that almost always contains two droplets in series. When prey is close, they spin their bolas in a circle, creating a cone-shaped space with which to strike their prey [33,34]. This genus appears less discerning to stimuli than *M. hutchinsoni* though, as they respond to human voice in addition to the wingbeats of prey, as seen in *M. hutchinsoni* [34,35,36]. *Cladomelea akermani* takes this indiscernibility even further and begins its prey capture technique without the stimuli of prey at all. They begin construction of a bolas of variable length, between one and four droplets, immediately at sundown. They then spin their bolas, rocking their body forward to generate and maintain momentum, for intervals of up to 15 min [37]. Thus, is seems clear that momentum of the droplet is crucial for attachment to the moth. Recent studies have shown that within superhydrophobic channels, similar to those likely to be found in the micromesh of the moth scales, fluids with higher viscosity are drawn more quickly through capillary systems than those with low viscosity [32]. The impact force pushes glue below the top of the scale line to the base cuticle creating a counter-intuitive benefit of high viscosity. This appears to be caused by air pockets between the surface roughness and the high viscosity liquid that lowers the frictional drag of the fluid as the cohesive forces of the fluid pull the bulk mass forward [32]. We look forward to future studies in Cyrtarachninae species that address the evolution of diverse biomechanical solutions for catching moths.

### 4.5. Caveats and Conclusions

Given our biomechanical predictions and evolutionary hypotheses, we want to acknowledge that the limited kinematic and mechanical results of this paper should be treated with caution. The kinematics are based on the 2D view of one camera; thus, any movements that are not in a plane parallel with that of the camera’s optical sensor will be underestimated in terms of the magnitude, but not frequency, of displacement, velocity, and acceleration. Furthermore, the sample size is limited to ten individuals, only four of which were recorded capturing moths; one individual was recorded twice, which yielded a total of five capture events. In addition, this work was conducted in one location over an eight-day period. A small sample size and a single location may have yielded a sample of behaviors that does not represent the population-level variance of this species well. In terms of modeling the glue droplet as a viscoelastic spring, this is a first approximation based on what are likely order-of-magnitude estimates of stiffness and damping.

However, in spite of the limitations of our quantitative results, we can be certain that our recorded behavioral observations offer new insights into the hunting of *M. hutchinsoni*. The bolas spider and its target moths have, as part of their repertoire, a six-stage capture interaction that we saw repeated in five of our six recorded events (Figure 1, Figure 3 and Figure 4)—(1) detecting a moth, (2) creating a bolas, (3) flicking the bolas at the next moth that approaches, (4) resisting the escape attempted by moth, (5) reeling in the bolas with the moth attached, and (6) subduing the moth. The other capture event showed the spider successfully using a trapline of dangling bolases to capture a moth. Thus, this species has at least two ways of catching moths with bolases, which are (1) active flicking and (2) passive snagging. It is also worth keeping in mind that only adult females use bolases; males and early juveniles grab moths directly from the air [16]. Thus, within *M. hutchinsoni* as a whole—females, males, and juveniles—we see a variation in behaviors that is likely tied to the on-going co-evolution with the moths that they attempt to capture.

## Figures and Tables

**Table 1:** Kinematics of spider, moth, and bolas during prey capture.

	Average ± Standard Deviation (N = 5)
Maximum moth speed (ms^−1^)	3.75 ± 3.09
Maximum spider speed (ms^−1^)	1.44 ± 0.98
Moth Kinematics	
Impact velocity (ms^−1^)	0.22 ± 0.17
Impact kinetic energy (μJ)	2.23 ± 2.65
Maximum kinetic energy (μJ)	710.15 ± 1165.4
Capture Kinematics	
Reeling rate (ms^−1^)	0.017 ± 0.007
Falling speed (ms^−1^)	0.266 ± 0.111
Duration of reeling (s)	2.25 ± 0.34
Distance from spider when dropping (cm)	1.21 ± 0.64
Silk Kinematics	
Droplet strain (ε)	5.95 ± 1.59
Radial silk strain (ε)	0.32 ± 0.15
Droplet spring constant (μNm^−1^)	10.61 ± 4.6 (N = 4)

**Table 2:** Bolas droplet acceleration and spring constants during a flick.

Video	Maximum Acceleration *a_max_* (ms^−2^)	Spring Constant *k* (μNm^−1^)
1	0.103	0.6
2	0.387	1.67
3	0.449	1.72
4	0.257	1.32

## Data Availability

Data used to create Figure 2 and Table 1 and Table 2 are available as Supplementary Materials for download. The Simulink MATLAB damper-spring model and code, used to create Figure 7, are also available to download as Supplementary Materials.

## Supplementary Materials

The following supporting information can be downloaded at: https://www.mdpi.com/article/10.3390/insects13121166/s1, Video S1: Trapline Hunting technique, *Mastophora hutchinsoni,* Video S2: Moth caught on Bolas—Standard, Video S3: Moth caught on Bolas—Not Giving Up.
